# Transmission dynamics of a linear *vanA*-plasmid during a nosocomial multiclonal outbreak of vancomycin-resistant enterococci in a non-endemic area, Japan

**DOI:** 10.1038/s41598-021-94213-5

**Published:** 2021-07-20

**Authors:** Yoshihiro Fujiya, Tetsuya Harada, Yo Sugawara, Yukihiro Akeda, Masako Yasuda, Ayako Masumi, Junichi Hayashi, Nobuhiro Tanimura, Yoshihiro Tsujimoto, Wataru Shibata, Takahiro Yamaguchi, Ryuji Kawahara, Isao Nishi, Shigeyuki Hamada, Kazunori Tomono, Hiroshi Kakeya

**Affiliations:** 1 Department of Infection Control and Prevention, Graduate School of Medicine, Osaka University, Suita, Osaka Japan; 2 Research Institute for Microbial Diseases, Osaka University, Suita, Osaka Japan; 3 Division of Microbiology, Osaka Institute of Public Health, Osaka, Osaka Japan; 4 Aijinkai Inoue Hospital, Suita, Osaka Japan; 5 Department of Infection Control Science, Graduate School of Medicine, Osaka City University, Osaka, Osaka Japan; 6 Laboratory for Clinical Investigation, Osaka University Hospital, Suita, Osaka Japan; 7 Present Address: Department of Infection Control and Laboratory Medicine, Sapporo Medical University School of Medicine, Sapporo, Hokkaido Japan

**Keywords:** Bacterial genes, Disease prevention, Public health, Clinical microbiology, Antimicrobial resistance

## Abstract

The spread of vancomycin-resistant enterococci (VRE) is a major threat in nosocomial settings. A large-scale multiclonal VRE outbreak has rarely been reported in Japan due to low VRE prevalence. We evaluated the transmission of vancomycin resistance in a multiclonal VRE outbreak, conducted biological and genomic analyses of VRE isolates, and assessed the implemented infection control measures. In total, 149 patients harboring VanA-type VRE were identified from April 2017 to October 2019, with 153 vancomycin-resistant *Enterococcus faecium* isolated being grouped into 31 pulsotypes using pulsed-field gel electrophoresis, wherein six sequence types belonged to clonal complex 17. Epidemic clones varied throughout the outbreak; however, they all carried *vanA*-plasmids (pIHVA). pIHVA is a linear plasmid, carrying a unique structural Tn*1546* containing *vanA*; it moves between different *Enterococcus* spp. by genetic rearrangements. VRE infection incidence among patients in the “hot spot” ward correlated with the local VRE colonization prevalence. Local prevalence also correlated with vancomycin usage in the ward. Transmission of a novel transferrable *vanA*-plasmid among *Enterococcus* spp. resulted in genomic diversity in VRE in a non-endemic setting. The prevalence of VRE colonization and vancomycin usage at the ward level may serve as VRE cross-transmission indicators in non-intensive care units for outbreak control.

## Introduction

Vancomycin-resistant *Enterococcus faecium* (VREfm) was first reported in 1988 and has rapidly spread around the globe^1^. Infections caused by vancomycin-resistant enterococci (VRE) have limited treatment options and are associated with higher mortality rates than those caused by vancomycin-susceptible enterococci (VSE)^2,3^. The rate of vancomycin resistance among *E. faecium* isolates from blood cultures is 17.3% in Europe and approximately 80% in the United States^4,5^. Although VRE outbreaks have been occasionally reported in Japan^6,7^, the Japan Nosocomial Infections Surveillance (https://janis.mhlw.go.jp/english/report/index.html) reported only 0.9% vancomycin resistance in all clinical isolates of *E. faecium* in 2018.

The acquisition of vancomycin resistance in *Enterococcus* spp. is mediated by several *van* gene clusters, of which *vanA* is the most common and confers high-level resistance to both vancomycin (minimum inhibitory concentration [MIC] = 64‒100 mg/L) and teicoplanin (16‒512 mg/L)^8^. The *vanA* cluster is typically harbored on transposon Tn*1546*, which is generally present on conjugative plasmids. Tn*1546* is located on Inc18-like, RepA_N, and pMG1-like plasmids^9–12^, which are highly transferable plasmids capable of transmitting vancomycin resistance. Hence, conjugative plasmid transfer is responsible for the widespread VanA-type vancomycin resistance among enterococcal populations^1,9,13^.

Whole genome sequencing (WGS) distinguishes isolates of a range of nosocomial pathogens. WGS has elucidated the transmission route and clonal spread of VREfm strains in certain VRE outbreaks^14–16^. Hence, WGS has become a powerful tool in VRE outbreak investigation. In a single center outbreak of carbapenem-resistant *Enterobacterales* (CRE), WGS-based plasmid analysis revealed the transfer of plasmids harboring *bla*_KPC_ among various bacterial species^17^; however, limited studies have focused on the transmission dynamics of *van*-plasmids between *Enterococcus* spp. Hence, the potential contribution of transferrable plasmids harboring *van* to the spread of vancomycin resistance remains uncharacterized.

Here, we report a large-scale, prolonged VanA-type VRE outbreak in Japan. This study aimed to elucidate the epidemiological and microbiological characteristics of this multiclonal and multispecies VRE outbreak at a single institution in a non-endemic area, as well as the transmission dynamics of vancomycin resistance determinants via conjugative plasmids during the outbreak. Subsequently, we evaluated the factors contributing to outbreak control.

## Results

### Outbreak description and characteristics of VRE-positive patients

In total, 149 patients were identified as having been colonized or infected with VRE from April 2017 to October 2019 (see Supplementary Fig. 1). The median age of these patients was 77 years, and 77/149 (52%) were males (see Supplementary Table 1). The median number of days of hospitalization prior to a positive VRE result was 25 days. One hundred and sixteen (78%) patients had a history of prior admission to the hospital, of which 112 (97%), including those who were VRE-positive at admission, had stayed in ward A. In total, 124 (83%) patients had chronic kidney disease and 109 (73%) were on dialysis. In addition, 111 (74%) patients had limitations in activities of daily living (ADLs). In particular, 47 (32%) patients were bedridden, and 82 (55%) were diaper users. Moreover, antimicrobial agents were administered to 145 (95%) patients within a year before the first isolation of VRE, and only 55 (37%) patients had used vancomycin. No fatality attributed to VRE infection was observed during the outbreak.

### Microbiology and surveillance data

**Figure 1:**
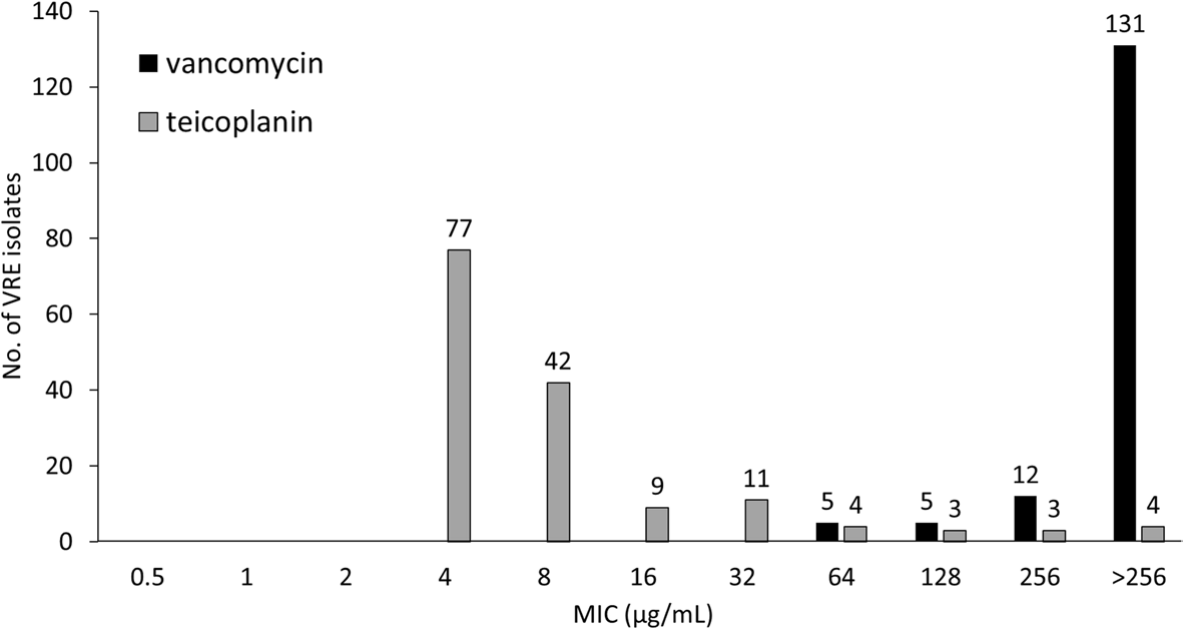
Minimum inhibitory concentration of vancomycin and teicoplanin for vancomycin-resistant *Enterococcus faecium* isolates during the outbreak. According to the criteria of the Clinical and Laboratory Standards Institute, breakpoints of vancomycin and teicoplanin are 4 µg/mL and 8 µg/mL, respectively (n = 153).

In total, 161 non-duplicated VRE isolates were recovered during the outbreak periods; 155 (96%) from 6720 stool samples or rectal swabs, 4 (2%) from urine, and 2 (1%) from wounds, of which 153 (96%), 4 (2%), 3 (2%), and 1 (1%) isolates were detected as *E. faecium*, *E. avium*, *E. raffinosus*, and *E. gallinarum*, respectively. All VRE isolates harbored *vanA*, whereas *E. gallinarum* harbored *vanC1* as well. All VRE isolates were resistant to vancomycin, and 131 (86%) VREfm isolates had an MIC > 256 µg/mL; however, 119 (78%) VREfm isolates had an MIC of 4 or 8 µg/mL for teicoplanin, indicating susceptibility to treatment according to the criteria of the CLSI (Fig. 1 and Supplementary Table 2).

### Analysis of clonality using PFGE and MLST

**Figure 2:**
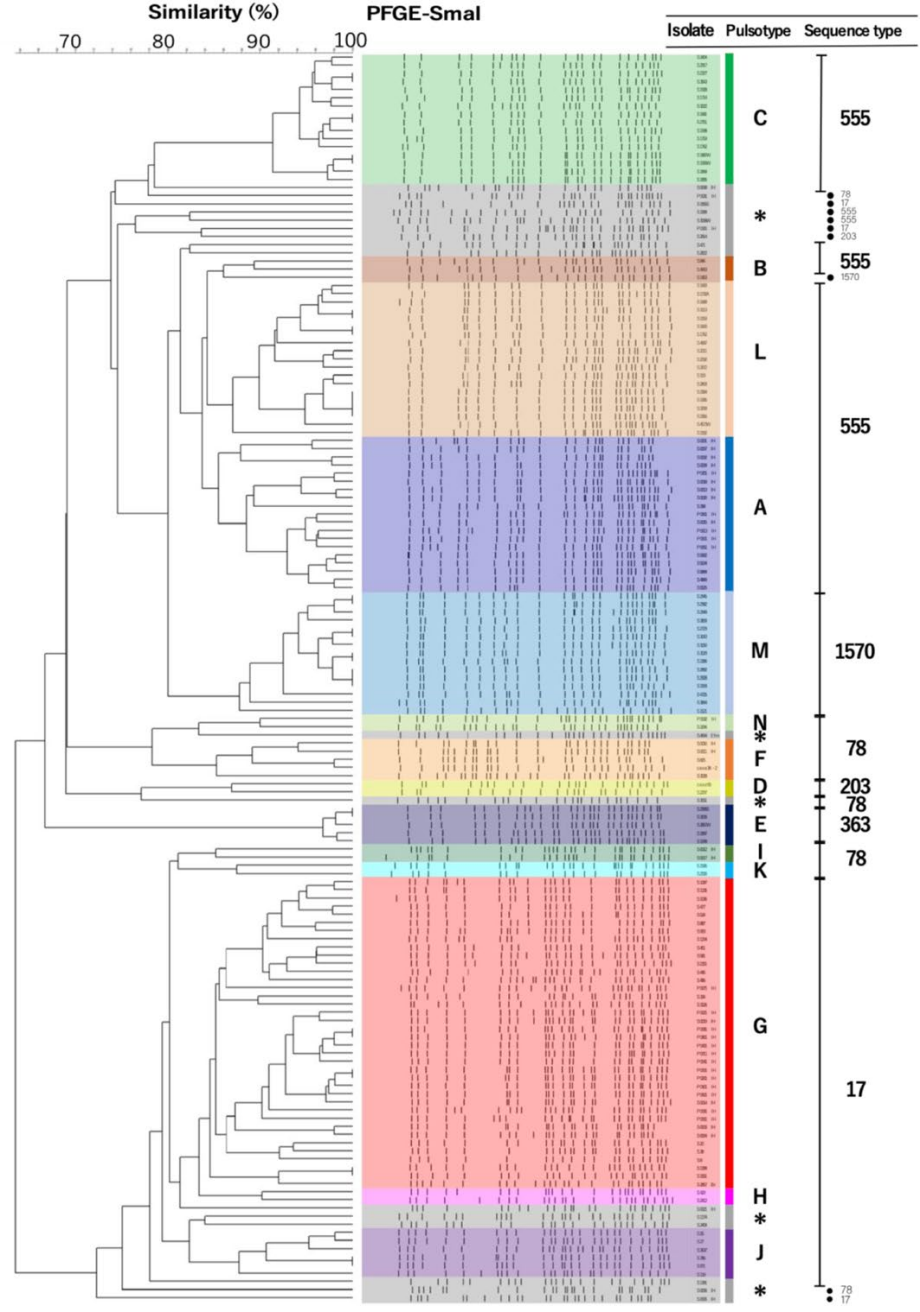
Dendrogram of pulsotypes in pulsed-field gel electrophoresis and sequence types in multilocus sequence typing among vancomycin-resistant *Enterococcus faecium* isolates (n = 153). *Singleton.

The PFGE analysis of 153 VREfm isolates identified 31 different patterns using a similarity cut-off of ≥ 85% (Fig. 2). In total, 132 (89%) isolates were classified into 14 pulsotypes, with pulsotype G being the most predominant (n = 38, 25%). In addition, four *E. avium* isolates showed different restriction patterns, whereas among the three *E. raffinosus* isolates, two were indistinguishable and one was closely related (see Supplementary Fig. 2). MLSTs of the VREfm isolates were divided into six sequence types (STs), ST17, ST78, ST555, ST203, ST363, and ST1530, all belonging to clonal complex (CC) 17 and hospital-adapted *E. faecium* (see Supplementary Fig. 3). All main pulsotypes, except for one isolate, corresponded to specific MLSTs (Fig. 2).

### Dynamics and diversity of VREfm

**Figure 3:**
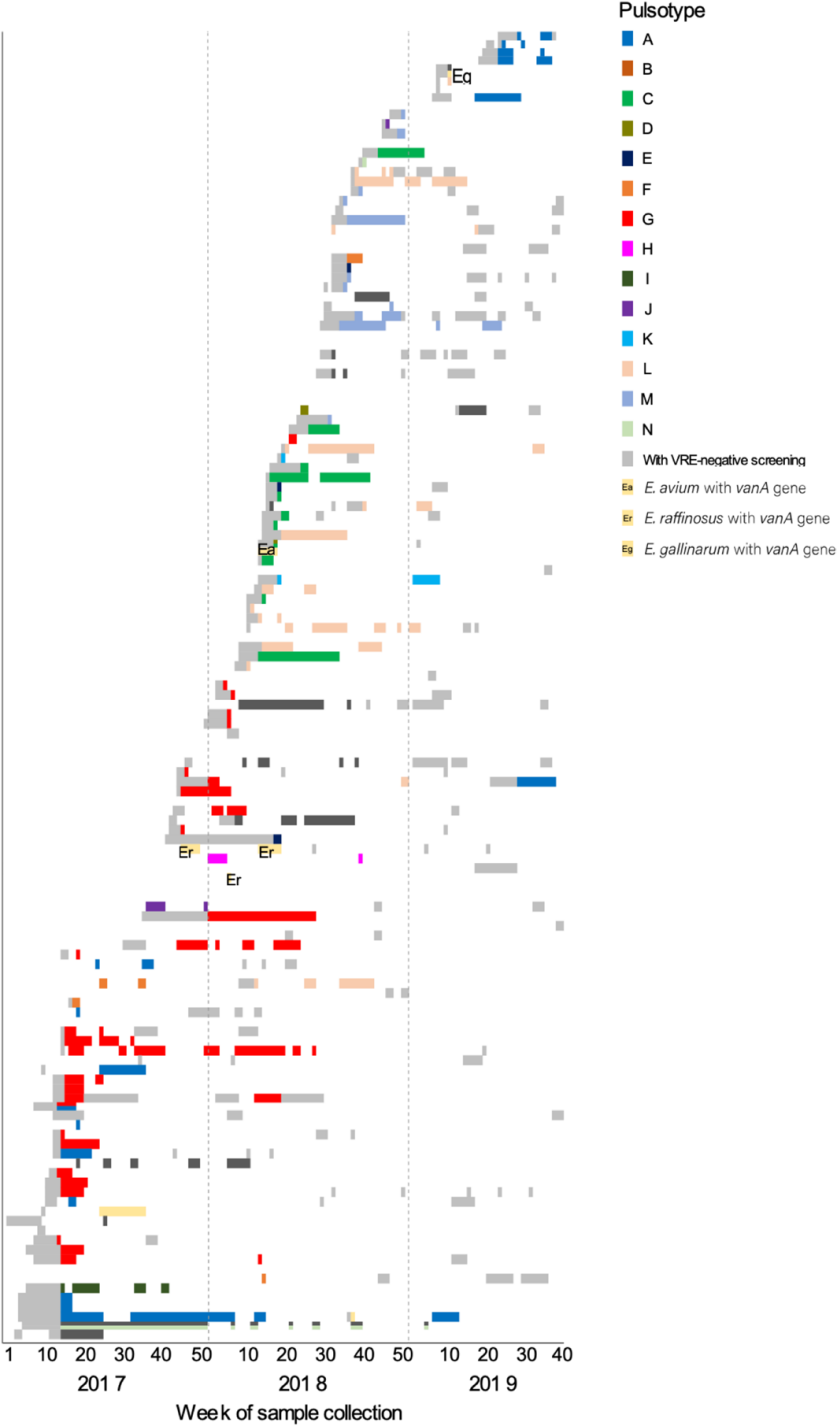
Temporal occurrence of vancomycin-resistant enterococci (VRE) and pulsotypes among VRE patients in ward A. Length of bar represents duration of hospitalization.

Temporal changes in the pulsotypes of VREfm and the length of stay in ward A for each patient are displayed in Fig. 3. Two main pulsotypes (A and G) and several minor pulsotypes were observed from 2017 until the first quarter of 2018 and subsequently disappeared. Pulsotypes C and L then appeared in the second quarter of 2018, while pulsotype M appeared in the second half of 2018. In 2019, horizontal spread of the pulsotype A-VREfm was triggered by readmission of a patient. These changes were similarly observed in wards B and C (see Supplementary Fig. 4).

### Mechanism of multiclonal outbreak in non-endemic settings

**Figure 4:**
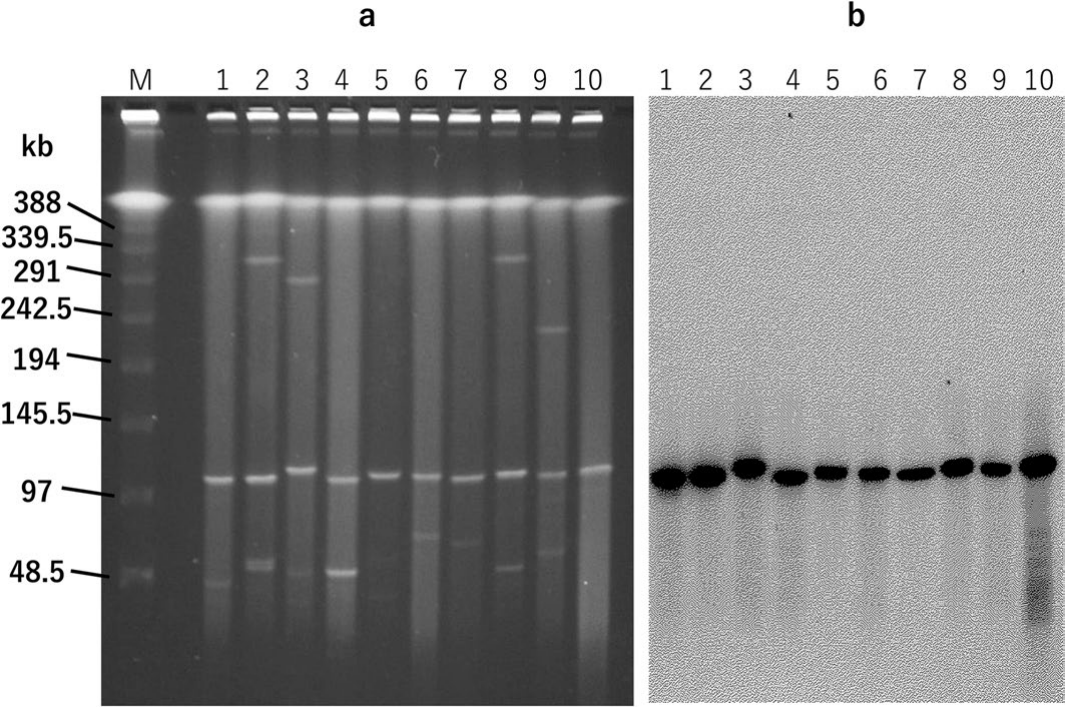
Pulsed-field gel electrophoresis of S1-digested plasmid DNAs from VanA-type vancomycin-resistant *E. faecium* isolates (**a**) and Southern hybridization with the *vanA* probe (**b**). (**a**) Lanes (left to right): M, molecular marker; 1, S209 (isolate), Er (strain), 10/2017 (date of sample collection), NA (pulsotype of Efm); 2, S398, Efm, 11/2017, A; 3, S477, Efm, 11/2017, G; 4, S844, Efm, 12/2017, B; 5, S1391, Efm, singleton; 6, S1693, Ea, 5/2018, NA; 7, S3088, Ea, 9/2018, NA; 8, S4573, Efm, 3/2018, L; 9, S4694Efm, Efm, 3/2018, singleton; 10, S4694Eg, Eg, 3/2018, NA. Er, *E. raffinosus*; Efm, *E. faecium*; Ea, *E. avium*; Eg, *E. gallinarum*; NA, not applicable. (**b**) Lanes (left to right): same as (**a**).

We performed S1-PFGE and Southern hybridization with a *vanA* probe for ten representative VRE isolates. The isolates were found to possess a plasmid of approximately 110 kb harboring *vanA* (Fig. 4), suggesting that the multiclonal outbreak was caused by transmission of this *vanA*-plasmid, termed pIHVA, between *Enterococcus* spp.

### Structural characteristics of pIHVA and Tn*1546*

**Figure 5:**
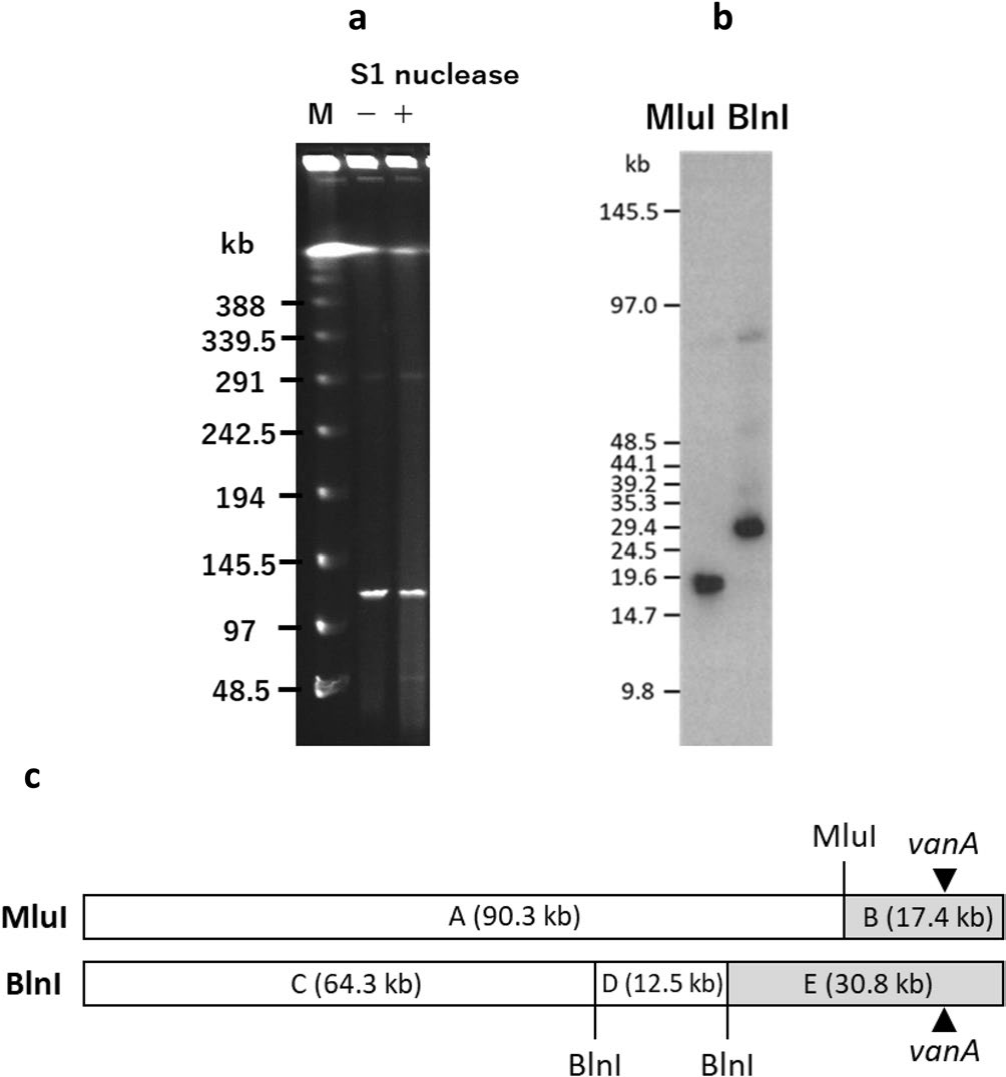
Molecular characterization of pIHVA, a linear plasmid. (**a**) Pulsed-field gel electrophoresis of S1 nuclease-treated and -untreated DNA of the vancomycin-resistant *E. faecium* S477 strain. M, molecular marker; +, S1 nuclease-treated; −, S1 nuclease-untreated. (**b**) Southern hybridization analysis of MluI and BlnI-treated DNA of the S477 strain with the *vanA* probe. (**c**) Restriction map of pIHVA (107 kb) is shown. A single MluI site and two BlnI sites are present in pIHVA. The fragments produced by digestion with these restriction enzymes are denoted by letters. The full-length gel and blot are presented in Supplementary Fig. 5.

A representative isolate Hb35 of pulsotype G and ST17 was selected for WGS. The genome of Hb35 included a single circular chromosome (2.86 Mb). Interestingly, the *vanA* cluster-harboring plasmid pIHVA-Hb35 (107,623 bp in size) was not circular. The electrophoretic mobility of pIHVA during PFGE analysis was comparable with or without the addition of S1 nuclease, which functions to nick supercoiled plasmid-DNA (Fig. 5a). In addition, the total DNA of the strain S477 containing the pIHVA plasmid identical to the pIHVA-Hb35 was digested with MluI and BlnI, followed by PFGE and Southern hybridization with a *vanA* probe. Positive signals were observed at approximately 17 Kb and 30 Kb on the digests from each enzyme (Fig. 5b,c). These were consistent with the estimated size of the restriction fragments obtained from the sequence data. Therefore, pIHVA-Hb35 was determined to be a linear plasmid.

**Figure 6:**
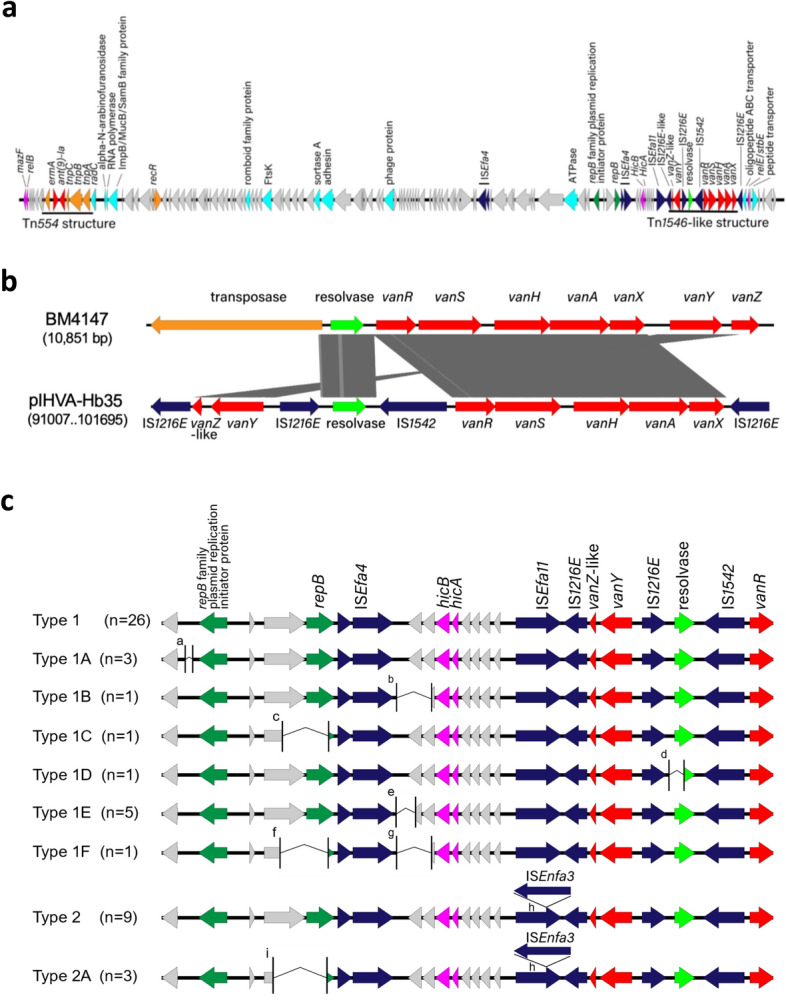
Genomic analysis of a linear plasmid carrying *vanA* (pIHVA). (**a**) Genomic structure of pIHVA from the Hb35 strain. Block arrows indicate confirmed or putative open reading frames (ORFs) and their orientations. Arrow size is proportional to the predicted ORF length. Color code is as follows: red, antimicrobial resistance genes; orange, transposase genes; green, plasmid replication initiator genes; light green, resolvase genes; cyan, genes encoding proteins of known function; deep blue, insertion sequence; and magenta, plasmid stabilization system. Putative, hypothetical, and unknown genes are represented by gray arrows. (**b**) Comparison of Tn*1546*-like region of pIHVA-Hb35 with its prototypical structure from BM4147. (**c**) Types of pIHVA found among 50 representative VRE isolates. ^a^Region deletion of 123 bp. ^b^Region deletion of 971 bp. ^c^Region deletion of 1400 bp. ^d^Region deletion of 1416 bp. ^e^Region deletion of 292 bp. ^f^Region deletion of 1380 bp. ^g^Region deletion of 944 bp. ^h^Insertion of IS*Enfa3* within IS*Efa11*., ^i^Region deletion of 1721 bp.

pIHVA-Hb35 contains a Tn*1546*-like structure comprising *vanA* (Fig. 6a). Compared with the Tn*1546* prototype BM4147 (GenBank accession no.: M97297), pIHVA-Hb35 did not contain a transposase gene; an inverted *vanYZ* was inserted upstream of resolvase, and *vanZ* was disrupted by IS*1216E* (Fig. 6b). pIHVA-Hb35 also contained a Tn*554* transposon, which carries *ermA* and *ant(9)-Ia* for macrolide- and spectinomycin resistance, respectively (Fig. 6a). In addition, major plasmid replication initiator genes (*rep* genes) and relaxase genes typically present on the *vanA*-plasmid were not detected on pIHVA-Hb35. The major plasmid stabilization systems, such as *axe-txe* and ω-ε-ζ, frequently identified in plasmids carrying *van*, were also not detected.

### Structural diversity of pIHVA

WGS revealed the presence of nine pIHVA variants in 50 representative VRE isolates (Fig. 6c). Type 1 was the most dominant (n = 26, 52%) and represented the backbone structure of pIHVA. Types 1A to 1F harbored one or two deletions within the type 1 structure. Type 2 was the second most dominant (n = 9, 18%) and had an insertion of IS*ENfa3* on IS*Efa11* within the type 1 structure. Type 2A exhibited a deletion within the type 2 structure. We observed the same pulsotype or ST of VREfm with different types of pIHVA and different pulsotypes or STs of VREfm with the same type of pIHVA (Supplementary Table 4). This indicates that pIHVA was transmitted between the endemic nosocomial *Enterococcus* spp. during the outbreak.

### Transmission of pIHVA among VRE

**Figure 7:**
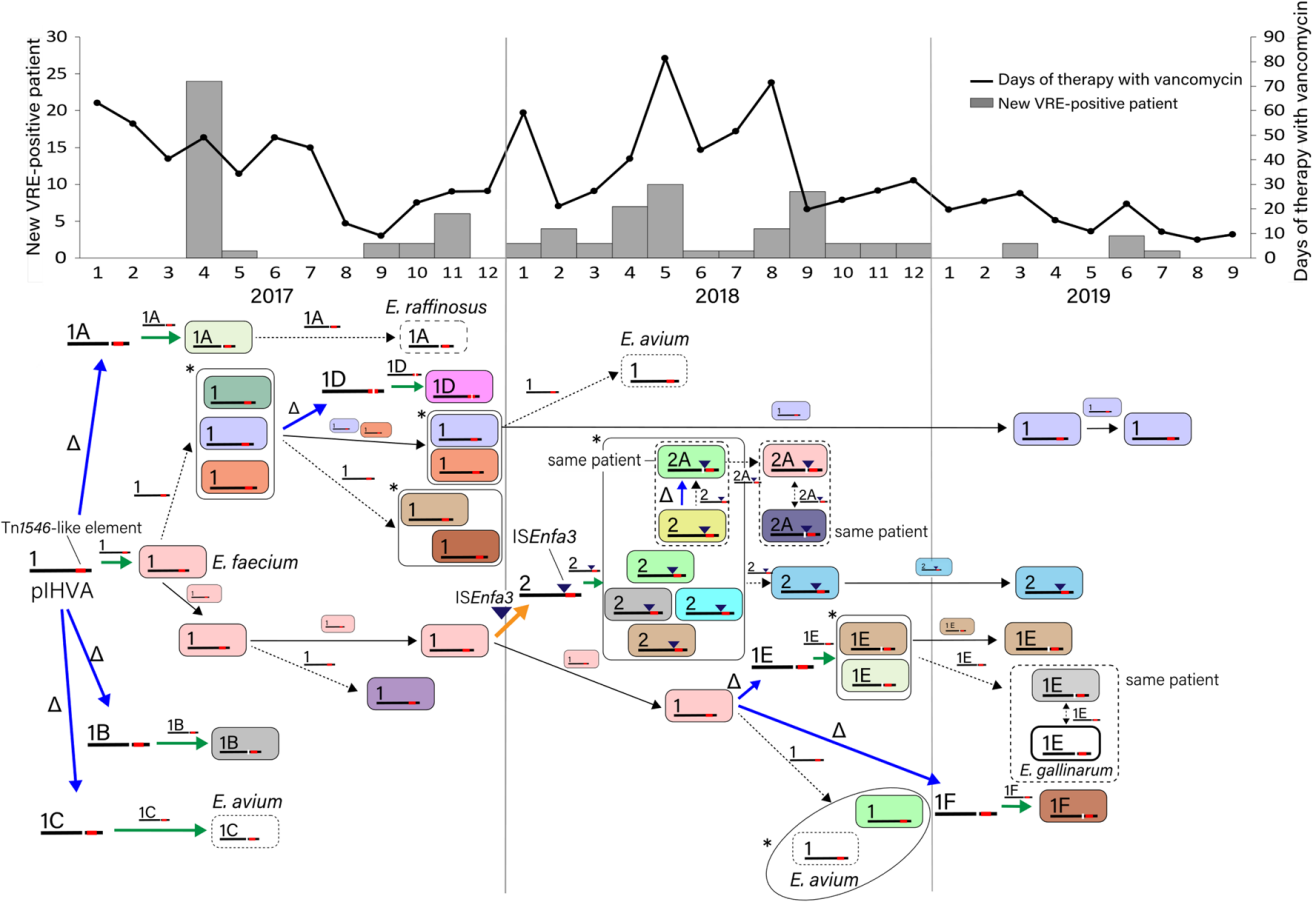
Transmission dynamics of a linear plasmid carrying *vanA* (pIHVA), monthly new vancomycin-resistant enterococci (VRE) patients, and days of therapy (DOT) with vancomycin in ward A. Time course in the dynamics corresponds to that of the graph. The color of the isolate indicates the pulsotype of PFGE as shown in Fig. 2. The number on the linear plasmids represents the type of pIHVA. Epidemiological investigation, microbiological examination, and genetic analysis of the plasmids indicated that pIHVA introduced into this hospital spread dynamically. This caused a multiclonal outbreak through genetic rearrangements via horizontal transmission of strains and plasmid transfer between strains. Vancomycin use was high in ward A during the period when diverse VREs were detected. Arrows: black, clonal spread; black dot, transmission of pIHVA; blue, deletion of a region on pIHVA; orange, insertion of IS*Enfa3*; green, uptake of pIHVA. Δ: deletion. *Enterococcal strains harboring the same type of *vanA*-plasmid with epidemiological links.

The dynamics of VRE dissemination was assessed in accordance with pulsotypes and pIHVA transmission (Fig. 7). pIHVA-1 plasmids were primarily recovered from a few pulsotypes of VREfm. Simultaneously, type 1A, 1B, and 1C plasmids were also recovered and were taken up by *Enterococcus* spp. From September to December 2017, new pulsotypes of VREfm with the type 1 plasmid appeared, most of which were detected during the admission screening. In 2018, type 2 plasmid was recovered from many pulsotypes of VREfm in ward A, followed by type 1 plasmid from different pulsotypes of *E. avium* and type 2A and 1E plasmids from several pulsotypes of VREfm. These results indicate that pIHVA was frequently disseminated by transmission between *Enterococcus* spp. over this period. Interestingly, heavy usage of vancomycin was observed in ward A in this period. In 2019, the transmission of pIHVA became less frequent.

### Factors contributing to outbreak control in ward A

**Figure 8:**
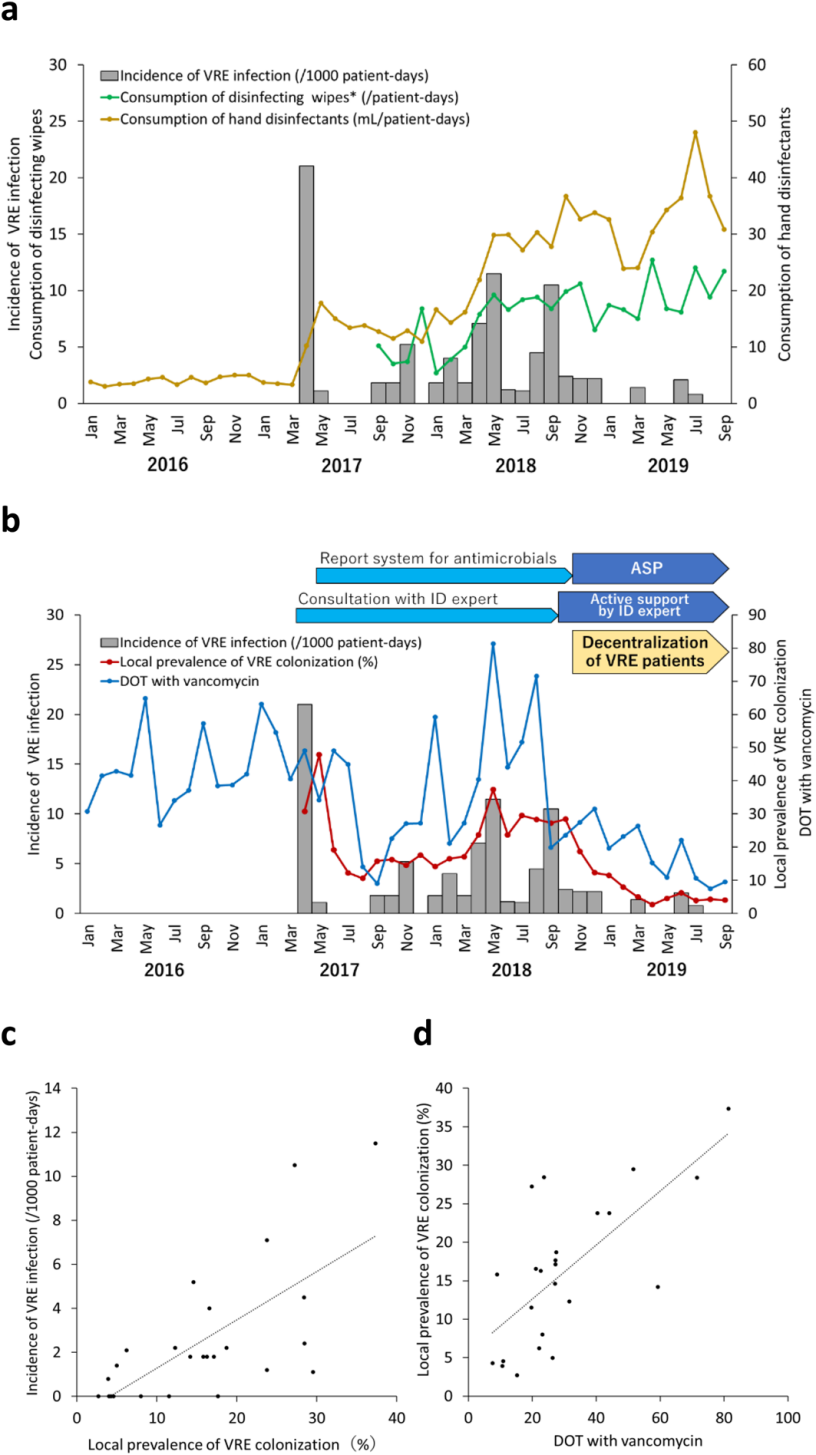
Incidence of vancomycin-resistant enterococci (VRE) infection and implemented infection control measures in ward A. (**a**) Consumption of hand disinfectants and disinfecting wipes. *Data before September 2017 were not available. (**b**) Local prevalence of VRE colonization and days of therapy (DOT) with vancomycin. *ASP* antimicrobial stewardship program, *ID* infectious disease. (**c**) Correlation between the incidence of VRE infection and local prevalence of VRE colonization. (**d**) Correlation between local prevalence of VRE colonization and DOT with vancomycin.

During the time period starting from the recognition of the outbreak in 2017 and May 2018, the utilization of hand disinfectants and disinfecting wipes gradually increased and IC measures were stringently followed in ward A, a “hot spot”; however, the outbreak was not controlled (Fig. 8a). Next, we analyzed the local prevalence of VRE colonization in ward A as patients undergoing dialysis tended to be frequently readmitted and patients with VRE were centralized to ward A as a strategy to prevent VRE outbreaks in other wards. We postulated that VRE was frequently transmitted under high VRE prevalence conditions. The median prevalence of VRE in ward A from September 2017 to December 2018 was 18.2%, which was significantly higher than that in wards B and C (6.1% and 3.1%, respectively; p < 0.01). However, in 2019, the prevalence in ward A significantly decreased to a median of 5.0% (p < 0.01). The incidence of VRE infection in ward A patients correlated well with the local prevalence (ρ = 0.62, 95% confidence interval [CI] 0.29–0.82, p < 0.01, Fig. 8b,c). In addition, the local prevalence was well correlated with DOT-VAN throughout ward A (ρ = 0.66, 95% CI 0.36–0.84, p < 0.01, Fig. 8b,d). These data indicated that high local prevalence of VRE colonization could be one of the factors for persistence of the outbreak and could be affected by ward-level vancomycin usage. In November 2018, preventive measures including decentralization, as well as an antimicrobial stewardship program by active support by an expert of infectious diseases, were introduced in ward A. In 2019, local prevalence, use of ward-level vancomycin, and incidence of VRE infection were significantly decreased; hence, we declared that the outbreak was terminated in October 2019.

## Discussion

In this study, we provide an overview of a multiclonal VanA-type VRE outbreak in a hospital specializing in dialysis therapy in a non-endemic area. Genomic analysis of the plasmids revealed the transmission dynamics of the *vanA*-linear plasmid and genomic rearrangements associated with it among *Enterococcus* spp., which contributed to a multiclonal outbreak. Ward-level prevalence of VRE colonization and vancomycin usage may be key indicators of VRE outbreak control.

Previous epidemiological studies indicated that hemodialysis and chronic renal failure are the risk factors of VRE colonization^18,19^. Meta-analysis indicated that recent use of antimicrobials, including vancomycin, and prior hospitalization would increase the risk of VRE colonization among dialysis patients^20^. Moreover, several studies have demonstrated that the administration of antimicrobials resulted in substantial changes in the gut microbiota that facilitated VRE colonization and allowed VRE to be the predominant intestinal species for several months even after cessation of the antimicrobials in a mouse model and humans^21,22^. In our study, almost all VRE-positive patients had recently received antimicrobials before isolation of VRE. Therefore, we speculate that most hospitalized patients in our hospital had antimicrobial-mediated disruption of gut microbiota and were a high-risk population for VRE colonization.

All *E. faecium* isolates in our study belonged to CC17, with the ST78 lineage being predominant, which is largely consistent with previous reports^6,7^. Notably, multiple STs and pulsotypes were observed among the VRE isolates; that is, a multiclonal outbreak was observed at this institution. We propose several hypotheses to explain this. First, there were multiple introductions of VRE into the hospital. In 2015, our team prospectively researched the distribution of CRE and VRE among hospitalized patients in the medical area where our hospital is located^23^. Only two (0.1%) VRE isolates were recovered, indicating that multiclonal VREfm would have been less likely to be introduced into the hospital. Second, vancomycin resistance could be acquired via a conjugative plasmid or Tn*1546*-like element containing *vanA*. In our study, S1-PFGE and genomic analysis identified a similar *vanA*-plasmid, pIHVA, in various strains of different species at different times, supporting our hypothesis. However, there have been few reports of such outbreak cases in non-endemic areas. Considering the host factors and heavy use of antimicrobials in the hospital, we assume that pIHVA was transferred into various VSE populations in high-risk hosts, reflecting the multiclonality of VRE. However, lack of VSE data is one limitation of our study.

Herein, we have elucidated the transmission dynamics of pIHVA, the vehicle for Tn*1546*, during the outbreak, which contributed to the multiclonal VRE outbreak. Moreover, hybrid-assembled short- and long-read data mapped to the reference sequencing data could detect the variants of pIHVA. At the beginning of the outbreak, most VREfm carried the type 1 plasmid. It is likely that a successful VREfm clone carrying a type 1 plasmid was introduced into the hospital at a particular time, followed by clonal spread and transmission of the plasmid via horizontal transfer into pre-existing *Enterococcus* populations with or without genomic rearrangements in pIHVA during the outbreak. The present outbreak was too complex for the transmission of VRE to be elucidated only by epidemiological investigation using PFGE. Thus, plasmid analysis has the potential to serve as a better tool to understand transmission dynamics during a VRE outbreak.

PFGE and Southern hybridization analyses indicated that pIHVA is a linear plasmid^24^. To our knowledge, this is the first report describing a linear *vanA*-plasmid-mediated VRE outbreak. Linear plasmids have been identified in several bacteria, such as *Streptomyces*, *Nocardia*, and *Mycobacterium*^25–27^. Recently, Hashimoto et al*.* identified a transferrable linear plasmid, pELF1, carrying the *vanA* and *vanM* clusters of VREfm in Japan^28^. pIHVA-Hb35 is similar to pELF1 (70% coverage and 98% identity). pIHVA and pELF1 are likely novel, broad-host-range transferrable plasmids as their sequences are completely different from those of well-known plasmids, such as Inc18-, pRUM-, pMG1-, and pHTβ-like^9^. Additionally, pIHVA also carries *ermA* and *ant(9)-Ia* found in the Tn*554* cluster, a *Staphylococcus aureus* transposon^29^. There may be an interaction between methicillin-resistant *S. aureus* and VRE at the genetic level. Thus, further research on linear *vanA* plasmids is warranted.

pIHVA carried a unique variant of Tn*1546*, in which *vanZ* was disrupted by IS*1216E*. Previous studies have reported that *vanZ* is involved in teicoplanin resistance and that deletion of *vanZ* could result in the loss of teicoplanin resistance^30,31^. In our study, 78% of VREfm isolates were susceptible to teicoplanin according to the CLSI criteria. We assumed that loss of a functional VanZ protein might result in a low MIC of teicoplanin.

We evaluated the prevalence of VRE colonization as a ward-level indicator to investigate the factors leading to the persistence of VRE transmission in this outbreak, considering that readmission of VRE-colonized patients continuously introduced VRE into the hospital and these strains remained in the premises for several months. Previous studies focused on an individual-level risk that a patient would acquire VRE in the ICU^32–34^. However, we found a significant correlation between the prevalence and incidence of new VRE patients in non-ICU, general wards. For the infection control (IC) manager, this may be a potential indicator to predict cross-transmission of VRE in general wards because of its simplicity and clarity. In addition, the prevalence was well correlated with DOT-VAN in Ward A. Therefore, high DOT-VAN at unit level, which means that pathogens are under high selection pressure, may affect the prevalence of VRE colonization. Of note, these do not indicate a causal relationship and are only potential indicators for IC managers to predict the trend of an outbreak.

The consumption of alcohol-based disinfectants dramatically increased in ward A; however, VRE transmission was not controlled. The majority of hospitalized patients were elderly with low ADLs, for whom nursing care was associated with VRE colonization (Supplementary Table 3). We assume that the care actions required for the patients could have increased health-care worker-patient contact rates and workload, which could have led to relative overcrowding, understaffing, and low cohorting levels^35–37^. Thus, hand hygiene compliance might have been low despite the highest level of consumption of hand disinfectants.

Our study has several limitations. As this was conducted in a single and unique institution, the results may have some regional and institutional biases. We focused on VanA-type VRE and used the Enterococcosel agar containing 32 µg/mL of vancomycin for active screening considering that only VanA-type VRE was isolated at the beginning of the outbreak. Therefore, it is possible that VanB-type VRE could not be cultured by this method. Lastly, due to the associated cost and effort involved in sequencing, not all VRE isolates were analyzed. Hence, a higher variety of plasmid types might still be identified.

In conclusion, genomic analysis provided a better understanding of the transmission dynamics of the linear *vanA*-plasmid among *Enterococcus* spp. throughout the outbreak, which would contribute to diverse VRE clones in a non-endemic area. Microbiological and clinical significance of the linear *van*-plasmid should be further researched. A VRE outbreak like the one discussed in the present study would be very complex and challenging to control with conventional IC measures. Bed management and antimicrobial stewardship at the ward/institutional level may be the key strategies for controlling and preventing VRE outbreak.

## Methods

### Study setting, design, and ethics

The hospital chosen for this study is located in Osaka, Japan, maintains 127 beds, and primarily specializes in chronic kidney disease and dialysis. A large dialysis center is annexed to the hospital. The hospital consists of three wards: ward A for internal medicine and vascular surgery, ward B for community-based integrated care, and ward C for internal medicine and orthopedics. Patients undergoing dialysis tend to be admitted to this hospital and, if required, are frequently readmitted.

A nosocomial VRE outbreak is described retrospectively in this study. Epidemiological and clinical data were collected from medical charts and the hospital database. The Ethics Committee of Osaka University Hospital approved the study and waived informed consent based on the retrospective design of the study (approval number: 19232). All methods were carried out in accordance with relevant guidelines and regulations.

### Surveillance policy and sample collection

Active surveillance was initiated in September 2017 targeting all new admissions, discharged patients who stayed for > 1 week, and inpatients who had been staying > 1 month. Stool or rectal swab samples were collected. If VRE was isolated, the patients were cohorted with strict contact precautions and followed up with monthly screenings. If the monthly screening yielded negative results for three consecutive months, standard precautions were employed.

### Case–control study

We conducted a retrospective case–control study from October to November 2017. Cases were defined as all hospitalized patients during the above period with an initial negative result for VRE followed by a positive result. As additional requirements, a sample that tested positive for VRE was collected after day 4 of admission, and the patients had no prior history of isolation of VRE from any previous clinical or screening samples. Controls were defined as all hospitalized patients during the same period with an initial negative result for VRE followed by ≥ 1 negative result, with no incidence of isolation of VRE in any culture before their inclusion in the study. Cases were matched to controls at a 1:2 ratio.

### Microbiological analysis

Collected samples were inoculated onto Enterococcosel agar (Becton–Dickinson Japan, Tokyo, Japan) containing 32 µg/mL of vancomycin and incubated aerobically at 37 °C for 48 h. Positive cultures were subjected to matrix-assisted laser desorption ionization-time of flight mass spectrometry (MALDI Biotyper; Bruker Daltonik GmbH, Bremen, Germany) to identify the species. Susceptibility of VRE isolates to vancomycin and teicoplanin was tested using E-test (bioMèrieux, Durham, NC, USA) and was interpreted in accordance with the Clinical and Laboratory Standards Institute (CLSI) document M100 ED29. Types of *van* genes were confirmed through multiplex PCR, as previously described^38^.

### PFGE and Southern blot hybridization

SmaI digestion of total DNA from VRE isolates was performed in accordance with the manufacturer’s protocol (Nippon Gene, Toyama, Japan). PFGE was conducted on a 1.0% SeaKem Gold agarose gel (Cambrex Bio Science, Rockland, ME, USA) and a CHEF-DRIII apparatus (Bio-Rad Laboratories, Hercules, CA, USA) in 0.5 × Tris–borate-EDTA (TBE) buffer at 14 °C and 200 V. A linearly ramped switching time of 0.7–15 s was applied for 19 h.

XbaI-digested DNA of the PFGE standard strain *S. enterica* serovar Braenderup H9812 was used as a molecular marker. S1 nuclease (Takara Bio, Shiga, Japan) digestion of total DNA from VRE isolates was performed in accordance with the manufacturer’s protocol. PFGE was performed as described above. A linearly ramped switching time of 6.7–22.7 s was applied for 15 h. CHEF DNA size standard, 48.5–1000 kb Lambda ladder (Bio-Rad Laboratories, Hercules, CA, USA) was used as a molecular marker. To determine the restriction map of pIHVA, total DNA from VRE isolate S477 containing the type 1 pIHVA was separately digested at 37 °C for 1 h with 20 U of MluI (TOYOBO, Shiga, Japan) and at 37 °C for 1 h with 16 U of BlnI (Takara Bio, Shiga, Japan). These DNA samples were then subjected to PFGE as described above. CHEF DNA size standard 5 Kb ladder (Bio-Rad Laboratories, Hercules, CA, USA) was used as a molecular marker.

DNA fragments were then transferred to a nylon membrane, hybridized with a digoxigenin-labeled probe (Roche, Basel, Switzerland) specific to *vanA*, and detected with CDP-Star Chemiluminescent Substrate (GE Healthcare Life Science, MA, USA).

Among VREfm, digital images were analyzed using BioNumerics software, version 6.6. If a difference in the PFGE pattern was observed, a new pulsotype was assigned. The definition of a PFGE cluster was based on a similarity cutoff of 85% (Dice coefficient, represented by UPGMA, 1.0% optimization and 1.0% tolerance). In addition, the PFGE homology of *E. avium* and *E. raffinosus* was determined using Tenover criteria^39^.

### Multilocus sequence typing (MLST)

Seven house-keeping genes (*adk*, *atpA*, *ddl*, *gdh*, *gyd*, *purK*, and *pstS*) were amplified and sequenced using previously described forward and reverse primers^40^. Sequence types were determined by submitting these data to the MLST website database (http://efaecium.mlst.net/). Cluster analysis was performed using the goeBURST algorithm^41^.

### Genomic analysis

For genomic analysis of the *vanA*-plasmid (pIHVA), we selected one isolate, EV0426-12, recovered from the nozzle of a bidet and 50 more VRE isolates representing four species and different pulsotypes of VREfm at different times (Supplementary Table 4). The EV0426-12 plasmid sequence was obtained using short- and long-read sequencers. Genomic DNA was extracted using the DNeasy PowerSoil Kit (Qiagen, Hilden, Germany). Short-read sequencing was performed using the Illumina MiSeq system with a MiSeq 500 cycle v2 kit. Libraries were constructed using the KAPA HyperPlus Library Preparation Kit (Roche Sequencing and Life Science, Kapa Biosystems, Wilmington, MA, USA). Long-read sequencing was conducted using a Nanopore GridION sequencer (Oxford Nanopore Technologies, UK) with a Ligation Sequencing Kit 1D (SQK-LSK108, Oxford Nanopore Technologies, Oxford, UK) and Native Barcoding Kit 1D (EXP-NBD103, Oxford Nanopore Technologies, Oxford, UK). The reads were assembled and polished using Unicycler^42^ or CANU 1.8^43^. For the 50 selected isolates, including Hb35, the first isolate in this study and a representative of the largest pulsotype, bands corresponding to the 110 kb plasmid were cut from S1-PFGE gels after staining with SYBR Gold nucleic acid stain, and the DNA was extracted using MagExtractor (TOYOBO Life Science, Osaka, Japan) for Illumina sequencing. Library preparation was performed using Nextera DNA Flex Library Prep Kit (Illumina), and sequencing was conducted as described above. Sequence reads were mapped to the assembled plasmid sequence of EV0426-12 (DDBJ accession number: LC566215) using CLC Genomics Workbench 11.0.1 (CLC bio, Aarhus, Denmark) to construct putative plasmid structures that were used for comparative analyses. ResFinder 3.2^44^ and PlasmidFinder 2.0.1^45^ databases were used to identify antimicrobial resistance genes and plasmid replicon types, respectively. Sequences were annotated with RASTtk^46^ and manually through a National Center of Biotechnology Information (NCBI) BLAST search. A detailed analysis of the insertion sequence was performed using ISfinder^47^. The sequence of *E. faecium* BM4147 (GenBank accession number: M97297) was used as a reference for the analysis of Tn*1546*-like sequence^48^. Genomic structures were compared and depicted using EasyFig^49^. Plasmids similar to pIHVA were identified using a BLAST search against NCBI database.

### Accession number(s)

The sequence data and details of the sequenced samples, including the date and location of collection and source of specimen, were submitted to the DDBJ/GenBank/ENA database under BioProject number PRJDB9108.

### Definition of indicators for infection control

The incidence was defined as the monthly number of new VRE-positive patients per 1000 inpatient-days. To evaluate the compliance with hand hygiene recommendations, the total volume of alcohol-based hand disinfectants consumed in a month was calculated in each ward and was divided by the total number of patient-days in each ward. For compliance to environmental cleaning, the total numbers of disinfecting wipes, potassium peroxymonosulfate cleaning agents, and wipes immersed in quaternary ammonium compounds used in a month were determined for each ward and divided by the total number of patient-days per ward. Ward level vancomycin usage was quantified monthly as days of therapy (DOT) in each ward from January 2016 to September 2019^50^. In cases where vancomycin was dispensed intermittently (e.g., alternate-day or after-dialysis regimen), DOT was defined as the total number of distinct days vancomycin was dispensed to the patient^51^. The ward level (local) prevalence of VRE colonization was calculated as the overall proportion of the total number of VRE-positive patient-days to the total number of all monthly patient-days per ward.

### Statistical analysis

All data were analyzed using JMP^®^ 13 (SAS Institute Inc., Cary, NC, USA). Continuous, non-parametric data were analyzed using the Wilcoxon rank-sum test. Spearman’s rank-order correlation coefficient ρ was determined among the incidence of VRE infection, the prevalence of VRE colonization, and days of therapy with vancomycin (DOT-VAN) in Ward A. A two-tailed p-value of < 0.05 was considered statistically significant.

## Supplementary Information

Supplementary Information.

## Data Availability

The datasets analyzed during the current study are available from the corresponding author on reasonable request.
