# Supplementary material for: Transmission dynamics of a linear vanA-plasmid during a nosocomial multiclonal outbreak of vancomycin-resistant enterococci in a non-endemic area, Japan
Source: Sci Rep. 2021 Jul 20;11:14780. doi: 10.1038/s41598-021-94213-5 (PMC8292306; doi:10.1038/s41598-021-94213-5)
Supplement: Supplementary file 1 — Supplementary Information. [file 41598_2021_94213_MOESM1_ESM.docx]

**Transmission dynamics of a linear *vanA*-plasmid during a nosocomial multiclonal outbreak of vancomycin-resistant enterococci in** **a non-endemic area, Japan**

Yoshihiro Fujiya, M.D.^1,2#a^, Tetsuya Harada, Ph.D^3^, Yo Sugawara, Ph.D^2^, Yukihiro Akeda, Ph.D^1,2^*, Masako Yasuda, RN^4^, Ayako Masumi, Pharm.D.^4^, Junichi Hayashi, BS^4^, Nobuhiro Tanimura Ph.D^4^, Yoshihiro Tsujimoto, Ph.D^4^, Wataru Shibata, Ph.D^5^, Takahiro Yamaguchi, Ph.D^3^, Ryuji Kawahara, Ph.D^3^, Isao Nishi, Ph.D^6^, Shigeyuki Hamada, Ph.D^2^, Kazunori Tomono, Ph.D^1^, Hiroshi Kakeya, Ph.D^5^

^1^Department of Infection Control and Prevention, Graduate School of Medicine, Osaka University, Suita city, Osaka, Japan

^2^Research Institute for Microbial Diseases, Osaka University, Suita city, Osaka, Japan

^3^Division of Microbiology, Osaka Institute of Public Health, Osaka city, Osaka, Japan

^4^Aijinkai Inoue Hospital, Suita city, Osaka, Japan

^5^Department of Infection Control Science, Graduate School of Medicine, Osaka City University, Osaka city, Osaka, Japan

^6^Laboratory for Clinical Investigation, Osaka University Hospital, Suita city, Osaka, Japan.

^#a^Current Address: Department of Infection Control and Laboratory Medicine, Sapporo Medical University School of Medicine, Sapporo city, Hokkaido, Japan

***Corresponding author:**

Yukihiro Akeda

Research Institute for Microbial Diseases, Osaka University

E-mail: akeda@biken.osaka-u.ac.jp

Phone number: +81-6-6879-4254

**Supplementary** **Information**

**
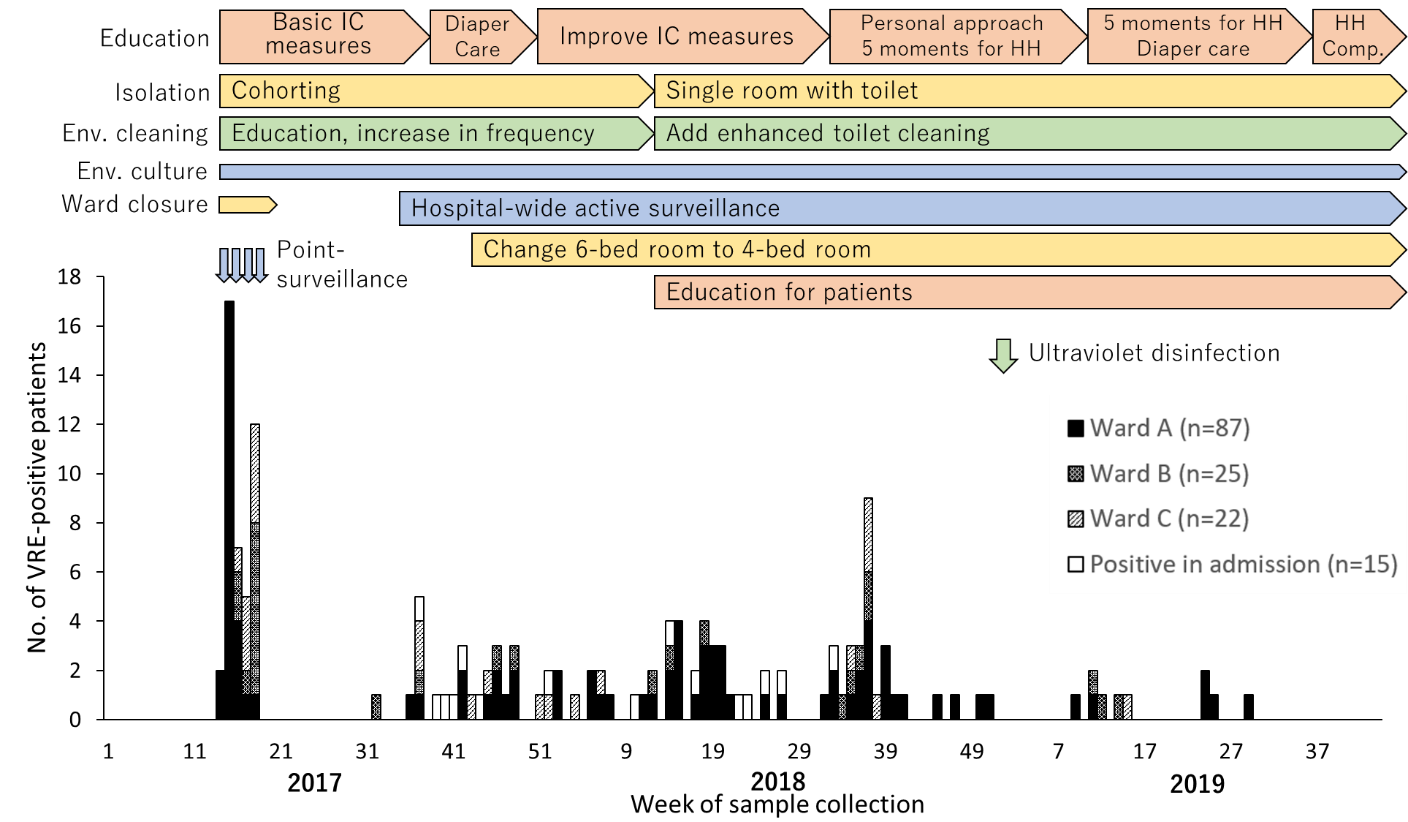
**

**Supplementary Figure 1.** Epidemiological chart and implemented infection control measures during the vancomycin-resistant enterococci outbreak in a hospital specializing in dialysis medicine in Japan (n = 149, 2017-2019). IC, infection control; HH, hand hygiene; Comp., compliance; Env., environment.

**A B**


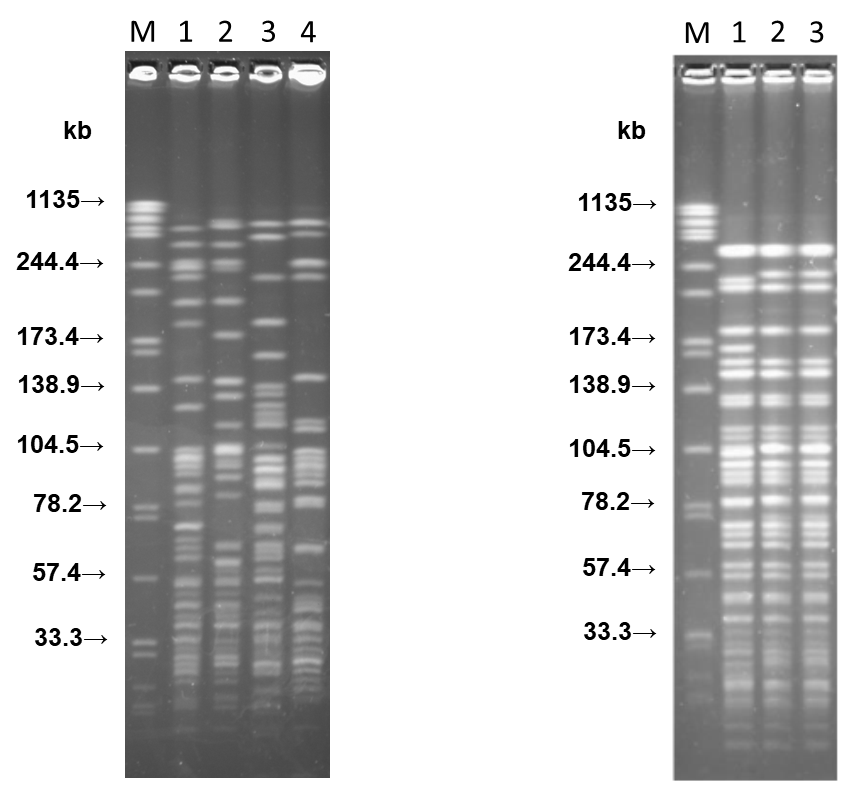


**Supplementary Figure 2.** Pulsed-field gel electrophoresis of SmaI-digested DNA from *Enterococcus avium* (A) and *Enterococcus raffinosus* (B) isolates. (A) Lanes (left to right): M, molecular marker; 1, S0016 (isolate), 5/2017 (date of sample collection); 2, S1693, 4/2018; 3, S2209, 6/2018; 4, S3088, 9/2018. (B) Lanes (left to right): M, molecular marker; 1, S209 (isolate), 10/2017 (date of sample collection); 2, case45, 9/2017; 3, S396, 11/2017. The full-length gels are presented in Supplementary Figure 6.


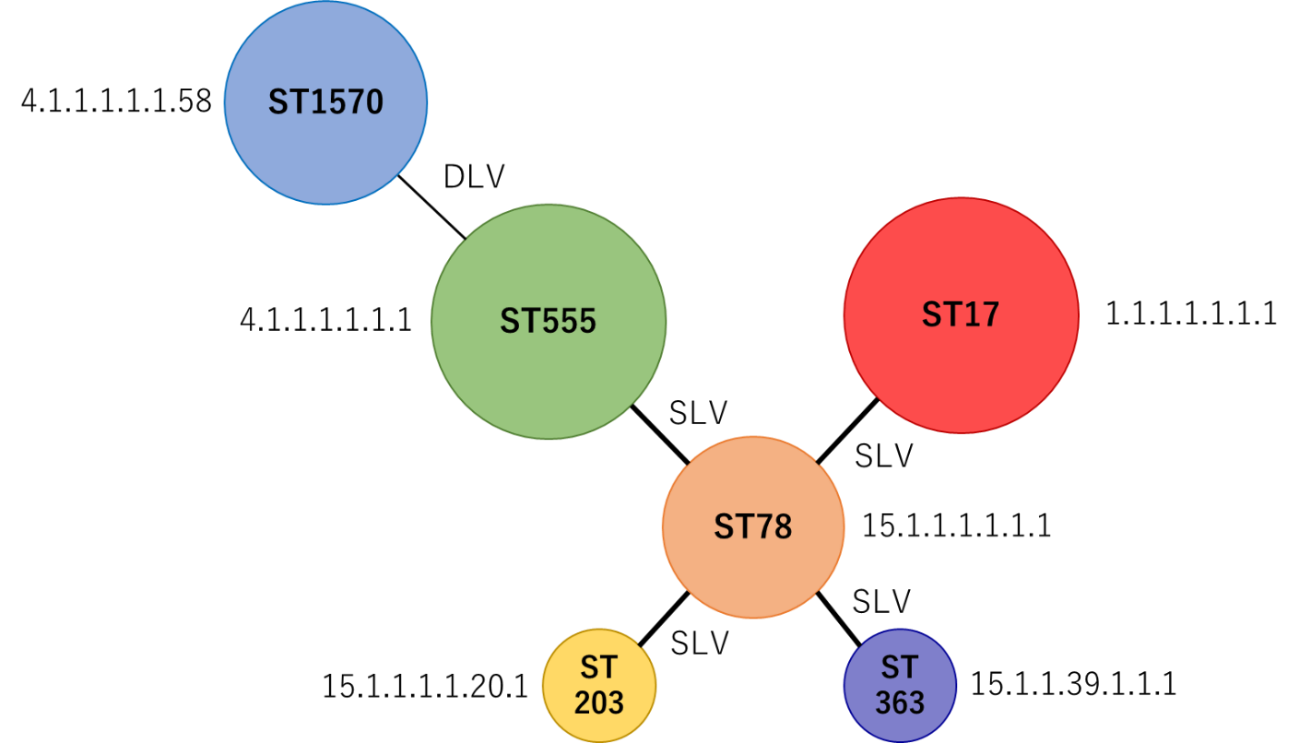


**Supplementary Figure 3.** eBURST analysis of the six sequence types (STs) identified among vancomycin-resistant *Enterococcus faecium* isolates. Each dot represents an ST, and the size of each dot corresponds to the number of isolates. The numbers represent the allele profiles and house-keeping genes (*atpA*, *ddl*, *gdh*, *purK*, *gyd*, *pstS*, *adk*) from multilocus sequence typing (MLST). SLV, single locus variant; DLV, double locus variant.

**ward B ward C**


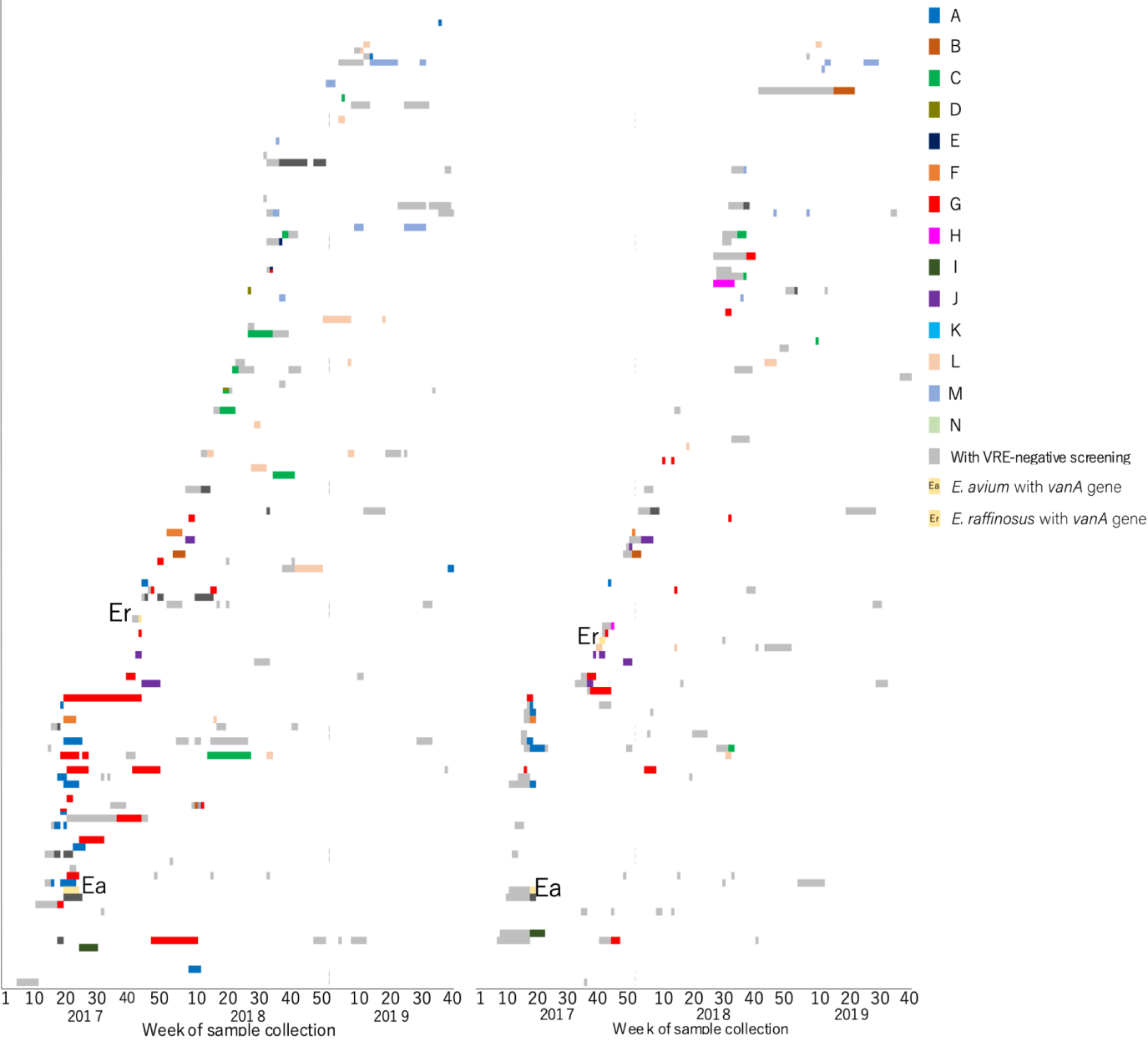
**Pulsotype**

**Supplementary Figure 4.** Temporal occurrence of VRE with pulsotypes from VRE patients in wards B and C. Colors indicate pulsotypes of VRE. Length of bar represents duration of hospitalization.

**A B**


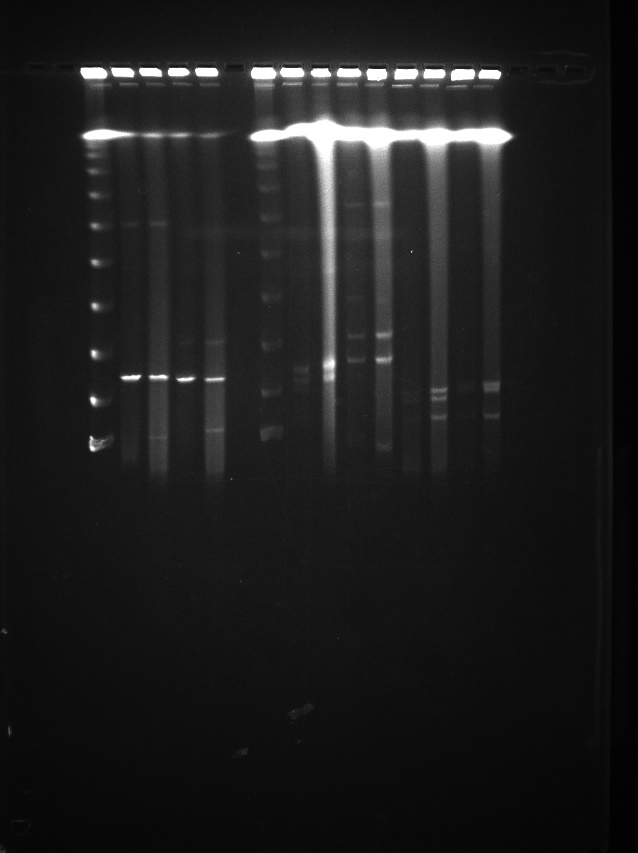

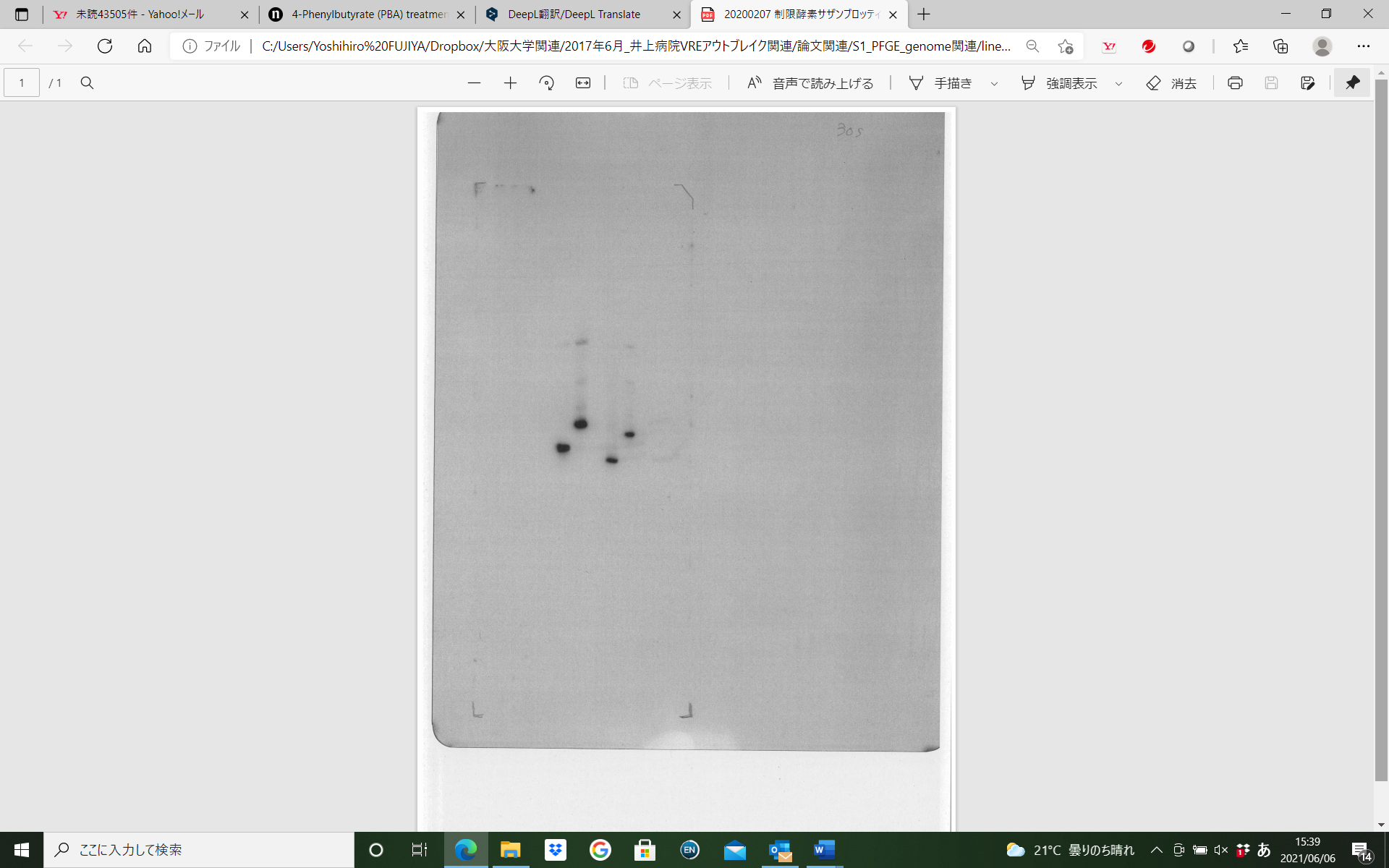


**Supplementary Figure 5.** The full-length gel and blot of Figure 5a (A) and 5b (B) of the manuscript (shown in the red boxes).

**A**


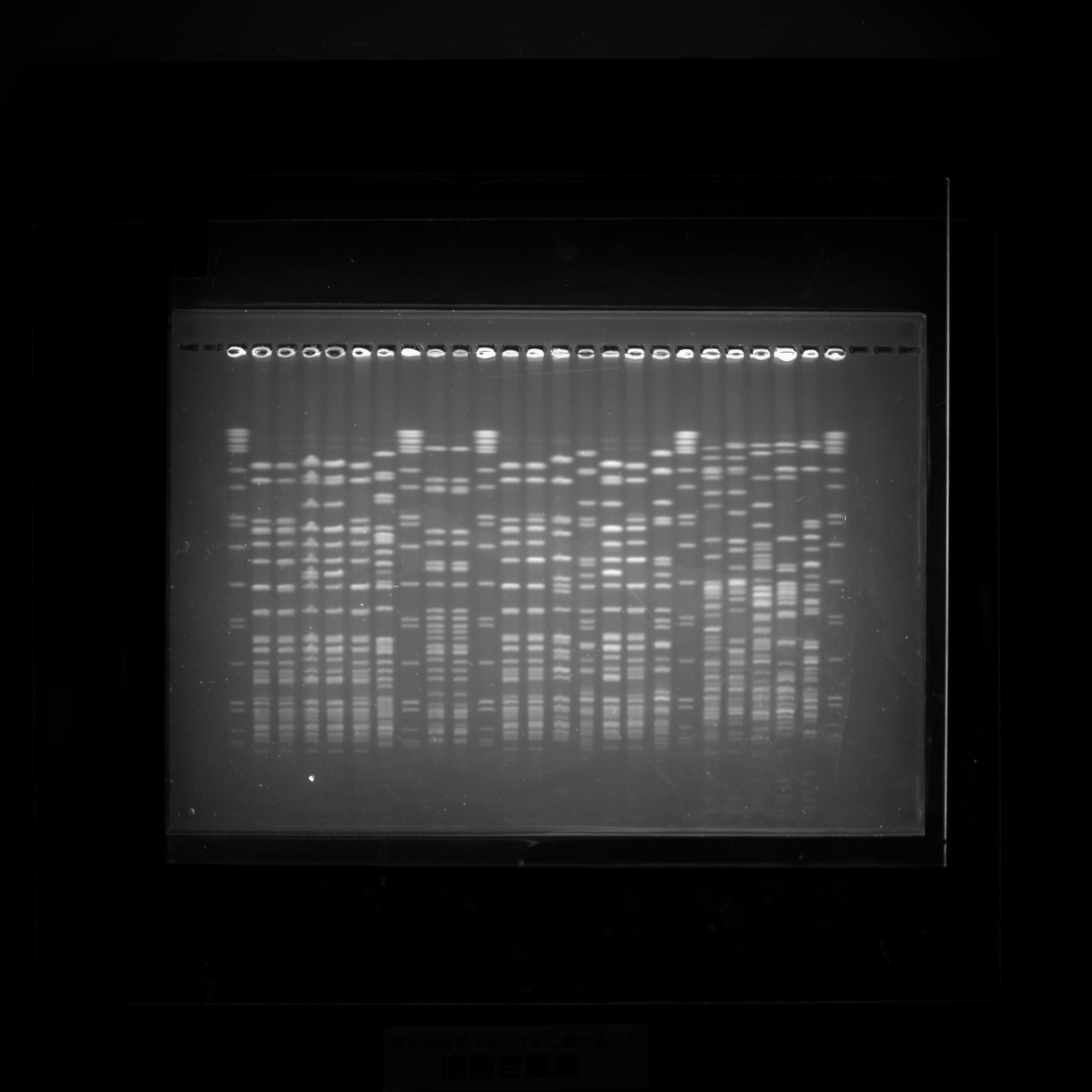


**B**


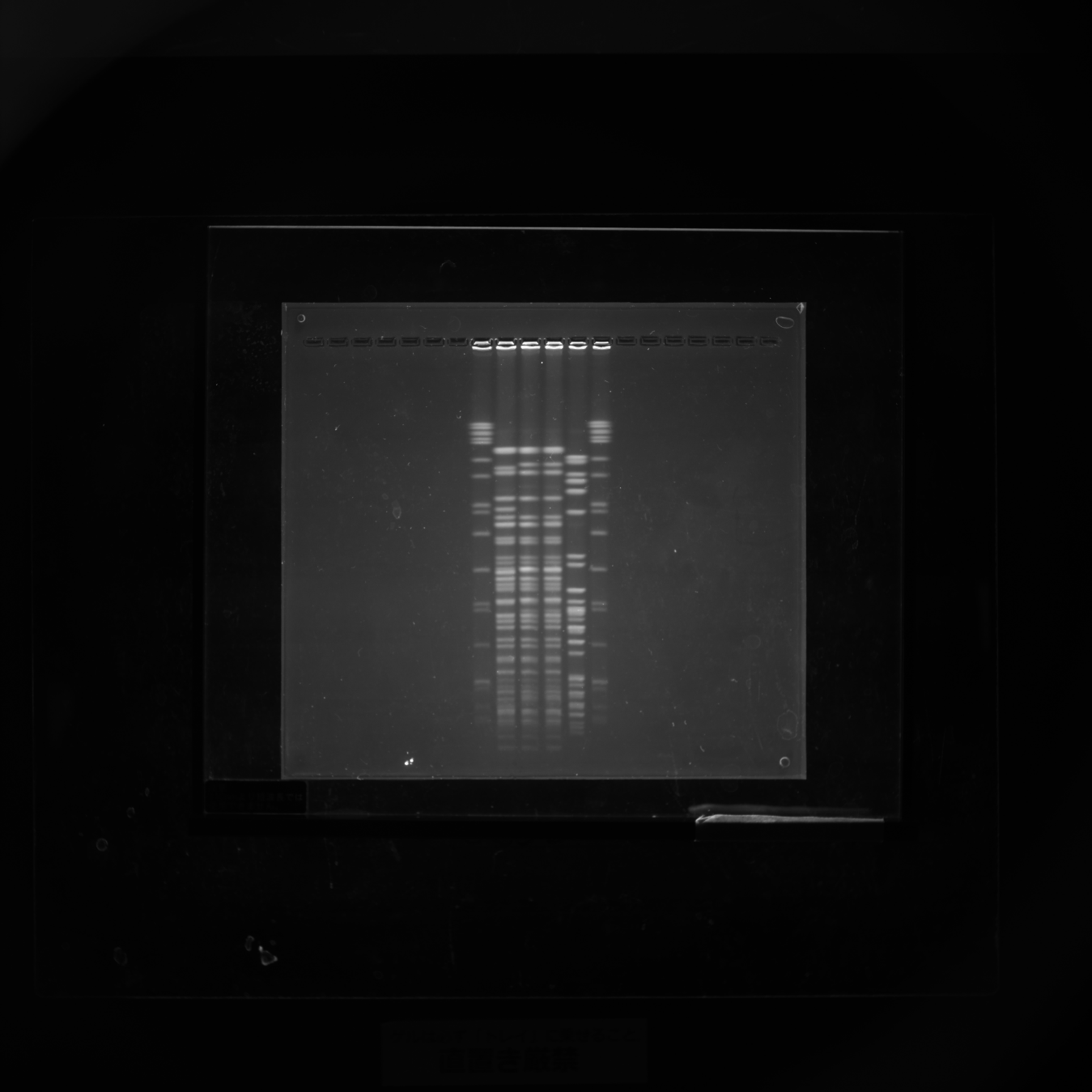


**Supplementary Figure 6.** The full-length gels of Supplementary Figure 2A (A) and 2B (B) (shown in the red boxes). We performed pulsed-field gel electrophoresis on our isolates, alongside ones from other cases.

**Supplementary Table 1.** Demographics of patients with vancomycin-resistant *Enterococcus faecium* infection during the outbreak (n = 149)

| Demographic | n (%) | Demographic | n (%) |
| --- | --- | --- | --- |
| Median age, year (IQR) | 77(69-84) | Nursing care, n (%) |  |
| Male, n (%) | 77 (52) | Oral care assist | 84 (56) |
| Specialty, n (%) |  | Genital cleaning | 82 (55) |
| Internal medicine | 98 (66) | Tracheal suction | 23 (15) |
| Vascular surgery | 28 (19) | Indwelling device, n (%) |  |
| Orthopedics | 18 (12) | Central venous catheter | 32 (21) |
| Surgery | 5 (3) | Urinary catheter | 19 (13) |
| Prior admission history, n (%) |  | Examination and Treatment, n (%) |  |
| Our hospital | 116 (78) | Endoscopy | 18 (12) |
| Ward A | 112 (75) | Operation | 51 (34) |
| Underlying disease, n (%) |  | Antimicrobial use during hospitalization | 133 (89) |
| Chronic kidney disease | 124 (83) | Vancomycin | 42 (28) |
| Dialysis | 109 (73) | Penicillin | 58 (39) |
| Diabetes mellitus | 57 (38) | Cephalosporins | 100 (67) |
| Peripheral arterial disease | 51 (34) | Carbapenems | 25 (17) |
| Cardiovascular disease | 38 (26) | Fluoroquinolones | 39 (26) |
| Cerebrovascular disease | 38 (26) | Any antimicrobial use within a year | 145 (95) |
| Dementia | 34 (23) | Vancomycin use within a year | 55 (37) |
| Foot gangrene | 28 (19) | Proton pump inhibitor use | 113 (76) |
| Activities of daily living, n (%) |  | Rehabilitation | 111 (74) |
| Bedridden | 47 (32) |  |  |
| Transfer assist | 64 (43) |  |  |
| Ambulant | 38 (26) |  |  |
| Diaper use | 82 (55) |  |  |
| Tube feeding | 9 (6) |  |  |

IQR, interquartile range

**Supplementary Table 2.** Distribution of minimum inhibitory concentrations of vancomycin and teicoplanin for vancomycin-resistant *Enterococcus* spp. isolates

| ***E. faecium* (n=153)** | | | | | | | | | | | |
| --- | --- | --- | --- | --- | --- | --- | --- | --- | --- | --- | --- |
| Antimicrobial agents | MIC (μg/mL） | | | | | | | | | | |
|  | 0.5 | 1 | 2 | 4 | 8 | 16 | 32 | 64 | 128 | 256 | >256 |
| vancomycin |  |  |  |  |  |  |  | 5 | 5 | 12 | 131 |
| teicoplanin |  |  |  | 77 | 42 | 9 | 11 | 4 | 3 | 3 | 4 |
|  | | | | | | | | | | | |
| ***E. avium* (n=4)** | | | | | | | | | | | |
| Antimicrobial agents | MIC (μg/mL） | | | | | | | | | | |
|  | 0.5 | 1 | 2 | 4 | 8 | 16 | 32 | 64 | 128 | 256 | >256 |
| vancomycin |  |  |  |  |  |  |  | 1 | 1 |  | 2 |
| teicoplanin |  |  |  | 2 |  | 1 |  |  |  |  | 1 |
|  | | | | | | | | | | | |
| ***E. raffinosus* (n=3)** | | | | | | | | | | | |
| Antimicrobial agents | MIC (μg/mL） | | | | | | | | | | |
|  | 0.5 | 1 | 2 | 4 | 8 | 16 | 32 | 64 | 128 | 256 | >256 |
| vancomycin |  |  |  |  |  |  |  |  |  |  | 3 |
| teicoplanin |  |  |  |  |  |  |  |  | 2 |  | 1 |
|  | | | | | | | | | | | |
| ***E. gallinarum* (n=1)** | | | | | | | | | | | |
| Antimicrobial agents | MIC (μg/mL） | | | | | | | | | | |
|  | 0.5 | 1 | 2 | 4 | 8 | 16 | 32 | 64 | 128 | 256 | >256 |
| vancomycin |  |  |  |  |  |  |  |  |  |  | 1 |
| teicoplanin |  |  |  |  |  |  | 1 |  |  |  |  |

MIC, minimum inhibitory concentration.

**Supplementary Table 3.** Case-control study among inpatients from September to November of 2017

| Variable | Patients, No. (%) | |  | Univariable  analysis^a^ | |  | Multivariable  analysis^b^ |
| --- | --- | --- | --- | --- | --- | --- | --- |
|  | Case (n=8) | Control (n=16) |  | P-value | OR (95% CI) |  | P-value |
| Median age, years | 77.0 | 76.5 |  | 0.31 | - |  |  |
| Days until VRE positive, days | 10.5 | 15.5 |  | 0.48 | - |  |  |
| Male sex | 4 (50) | 5 (31) |  | 0.41 | 2.20 (0.38-12.57) |  |  |
| Specialty |  |  |  |  |  |  |  |
| Internal medicine | 7 (88) | 13 (81) |  | 1.00 | 1.62 (0.14-18.58) |  |  |
| Admission |  |  |  |  |  |  |  |
| Prior history | 7 (88) | 12 (75) |  | 0.63 | 2.33 (0.22-25.24) |  |  |
| Ward A | 7 (88) | 11 (69) |  | 0.62 | 3.18 (0.30-22.36) |  |  |
| Room same as VRE-positive patient | 4 (50) | 4 (25) |  | 0.36 | 3.00 (0.50-17.95) |  |  |
| Underlying disease |  |  |  |  |  |  |  |
| Dialysis | 6 (75) | 10 (63) |  | 0.67 | 1.80 (0.27-11.96) |  |  |
| Diabetes mellitus | 2 (25) | 5 (31) |  | 1.00 | 0.73 (0.11-4.99) |  |  |
| Diarrhea | 6 (75) | 5 (31) |  | 0.08 | 6.60 (0.97-44.92) |  |  |
| Activity of daily living |  |  |  |  |  |  |  |
| Non-ambulant | 6 (75) | 5 (31) |  | 0.08 | 6.60 (0.97-44.92) |  | 0.99 |
| Diaper use | 6 (75) | 2 (13) |  | <0.01^*^ | 21.00 (2.37-185.92) |  | 0.99 |
| Tube feeding | 1 (13) | 1 (6) |  | 1.00 | 2.14 (0.12-39.47) |  |  |
| Nursing care |  |  |  |  |  |  |  |
| Oral care assist | 5 (63) | 3 (19) |  | 0.06 | 7.22 (1.08-48.47)^*^ |  | 0.99 |
| Cleaning in genial | 5 (63) | 3 (19) |  | 0.06 | 7.22 (1.08-48.47)^*^ |  | 0.99 |
| Tracheal suction | 3 (38) | 1 (6) |  | 0.09 | 9.00 (0.75-107.38) |  |  |
| Suppository insertion | 4 (50) | 1 (6) |  | 0.03^*^ | 15.00 (1.29-174.39) |  | 0.99 |
| Ice pillow use | 5 (63) | 2 (13) |  | 0.02^*^ | 11.67 (1.49-91.54) |  | 0.99 |
| Indwelling device |  |  |  |  |  |  |  |
| Central venous catheter | 2 (25) | 1 (6) |  | 0.25 | 5.00 (0.38-66.01) |  |  |
| Urinary catheter | 3 (38) | 1 (6) |  | 0.09 | 9.00 (0.75-107.38) |  |  |
| Examination and Treatment |  |  |  |  |  |  |  |
| Endoscopy | 3 (38) | 2 (13) |  | 0.29 | 4.20 (0.54-32.96) |  |  |
| Operation | 1 (13) | 8 (50) |  | 0.18 | 0.14 (0.01-1.44) |  |  |
| Antimicrobials use | 6 (75) | 7 (44) |  | 0.21 | 3.86 (0.59-25.29) |  |  |
| Prior antimicrobials use within a year | 6 (75) | 8 (50) |  | 0.39 | 3.00 (0.46-19.59) |  |  |
| Proton pump inhibitors use | 5 (63) | 8 (50) |  | 0.68 | 1.67 (0.29-9.45) |  |  |
| Rehabilitation | 6 (75) | 4 (25) |  | 0.03^*^ | 9.00 (1.27-63.89) |  | 0.99 |
|  |  |  |  |  |  |  |  |

OR, odds ratio; C.I., confidence interval.

^a^ Fisher's exact test or Wilcoxon rank-sum test was performed as appropriate.

^b^ Results of variables included in the model of multivariate analysis are shown.

* Statistically significant.

**Supplementary Table 4.** Characteristics of 50 representative VRE isolates analyzed using MiSeq sequencing
